# Non-*Helicobacter pylori* Gastric Intestinal Metaplasia in Children: A Series of Cases and Review of the Literature

**DOI:** 10.1155/2018/5930415

**Published:** 2018-04-19

**Authors:** Sandra Mabel Camacho-Gomez, Anas Bernieh, Ali G. Saad, Neelesh Ajit Tipnis

**Affiliations:** ^1^Department of Pediatrics, University of Mississippi Medical Center, 2500 North State Street, Jackson, MS 39216, USA; ^2^Department of Pathology, University of Mississippi Medical Center, 2500 North State Street, Jackson, MS 39216, USA

## Abstract

In the pediatric population, Gastric Intestinal Metaplasia (GIM) is a finding with unknown frequency and, more importantly, unknown clinical implications. The relationship between* Helicobacter pylori* (HP) infection and GIM is well documented, as well as an association between duodenogastric reflux and GIM. We present two cases of pediatric patients with GIM along with a review of the literature. The diagnosis of GIM may have adverse clinical implications and should be made with caution in a child. The association of GIM and adenoma/dysplasia and carcinoma is rarely seen in children, primarily because the time required for these to develop takes the individual into adulthood. Treatment, long-term consequences, and surveillance protocols are not well established in the pediatric population. Studies to evaluate the long-term natural history, treatment, and surveillance protocols in children with GIM are needed.

## 1. Introduction

Gastric Intestinal Metaplasia (GIM) is a finding with unknown frequency and, more importantly, unknown clinical implications in children. We present two cases of pediatric patients with GIM and a review of the literature. We discuss the epidemiology of GIM in patients with* Helicobacter pylori* (HP) gastritis and the potential role of HP gastritis and bile acid reflux in the development of GIM. We discuss histologic risk factors for the progression of GIM to gastric cancer. We also discuss the potential need for the long-term surveillance and natural history studies of GIM in children.

## 2. Case Report

### 2.1. Case 1

**Figure 1:**
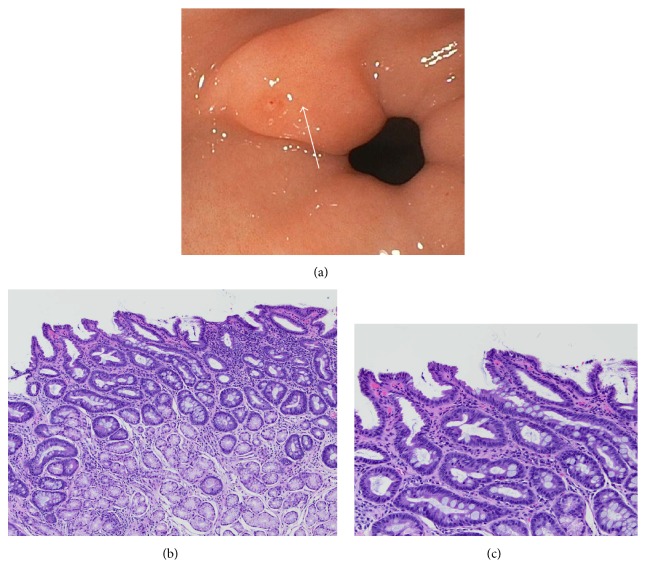
Endoscopic and histologic appearance of gastrointestinal metaplasia in case 1. (a) shows the appearance of a prepyloric nodule with an erosion located on the lesser curvature of the antrum. A cold forceps biopsy was taken from a region adjacent to the erosion (arrow). (b) shows a low power and (c) a high power histopathology image of the prepyloric nodule. The lamina propria is distended by a chronic inflammatory cell infiltrate consisting of lymphocytes and plasma cells. Numerous mucin producing cells, characteristic of intestinal epithelium, are identified. The features are those of chronic gastritis with intestinal metaplasia of the complete type.

A 16-year-old female presented to the clinic complaining of progressive worsening of dysphagia to solid foods with sensation of fullness in the chest and sour taste in the mouth but denies heartburn or chest pain. She did not have any weight loss. Her symptoms were unresponsive to PPI therapy started by her primary care physician. There was no family history of gastric cancer. The physical exam was unremarkable and the blood work was normal (including celiac panel, comprehensive metabolic panel, and HP IgG). An esophagram was normal. She underwent esophagogastroduodenoscopy (EGD), which revealed a 4 mm prepyloric nodule (see Figure 1(a)). A rapid urease test for HP was negative. Hematoxylin and eosin-stained sections on the prepyloric nodule biopsy showed antral-type gastric mucosa (see Figures 1(b) and 1(c)). The lamina propria was distended by a chronic inflammatory cell infiltrate consisting of lymphocytes and plasma cells. Numerous mucin producing cells, characteristic of intestinal epithelium, were identified. The features were those of chronic gastritis with intestinal metaplasia of the complete type. No dysplasia was present. No HP organisms were identified on light microscopy and by immunostain. Patient reports improvement in her symptoms after completing a course of double dose PPI therapy. Repeated EGD 1 year after showed resolution of the gastric erosion and persistence of the prepyloric nodule on EGD. Complete type intestinal metaplasia persisted on histologic evaluation.

### 2.2. Case 2

**Figure 2:**
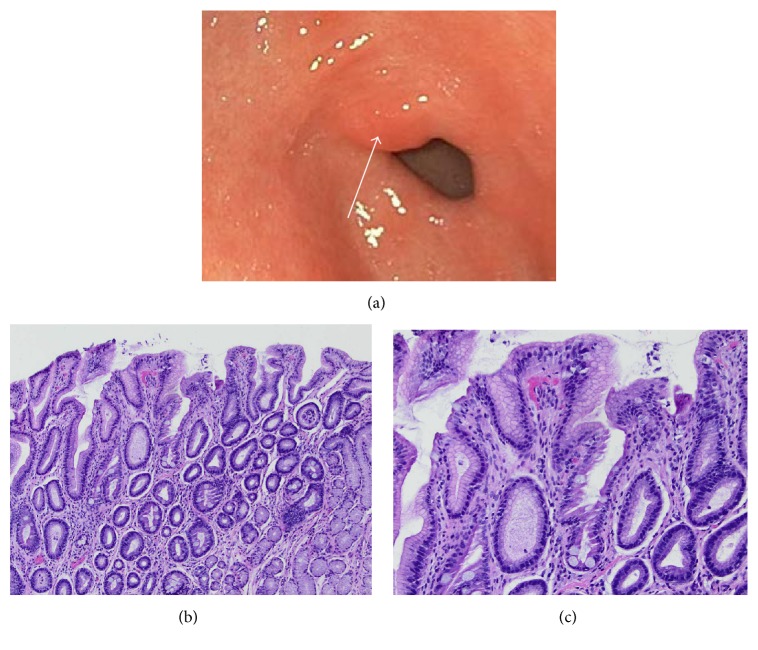
Endoscopic and histologic appearance of gastrointestinal metaplasia in case 2. (a) shows the appearance of a prepyloric nodule with an erosion located on the lesser curvature of the antrum. A cold forceps biopsy was taken from a region adjacent to the erosion (arrow). (b) shows a low power image and (c) shows a high power histopathology image of the prepyloric nodule in case 2. The lamina propria is distended by a chronic inflammatory cell infiltrate, irregular columnar cells filled with mucin, and an absence of brush border is noted, signifying an incomplete type.

An 8-year-old female with generalized but not radiated abdominal pain, described as cramping and sharp for the last one year, presented to our clinic. She reported intermittent nausea associated with nonbloody and nonbilious vomiting. Vomiting was more frequent at night. There was no family history of gastric cancer. The patient had an unremarkable blood work (including complete blood count, chemistry panel, antibodies to HP, and celiac panel). Computed tomography of the abdomen was significant for mesenteric adenopathy. The EGD showed a prepyloric nodule (Figure 2(a)) and bile-lake. Histological examination showed incomplete intestinal gastric metaplasia with irregular mucin droplets and an absent brush border (see Figures 2(b) and 2(c)). Rapid urease testing for HP, as well as immunostains, was negative. She was treated with double dose PPI therapy. Vomiting improved with cyproheptadine. Repeat EGD 6 months later showed resolution of the prepyloric erosion and continued presence of the prepyloric nodule. Histologic examination showed persistence of incomplete gastric intestinal metaplasia without progression to dysplasia.

## 3. Discussion

GIM is defined as the replacement of gastric columnar cells by cells of intestinal morphology characterized by the presence of mucin-containing goblet, Paneth, and absorptive cells [1]. The intestinal cells are easily distinguished in the gastric mucosa, because they are not present in healthy gastric mucosa [2]. The histopathologic diagnosis of GIM has been found to have high interobserver agreement [3, 4]. No consensus is available about the optimal number or location of biopsies needed in children [5]. In adults, biopsy mapping of the stomach requires at least 5 biopsy specimens: 2 from the antrum within 2 to 3 cm from the pylorus (1 each from the lesser and greater curvatures); 2 from the corpus approximately 8 cm from the cardia (1 each from the lesser and greater curvatures); and 1 from the incisura angularis [6].

The prevalence of GIM in children is largely unknown [7]. Furthermore, endoscopic features of GIM in pediatric patient are poorly defined. A white opaque substance visualized by magnifying endoscopy with narrow-band imaging (M-NBI) appears to be a useful indicator of the histological diagnosis of GIM [8]. GIM is a common finding on routine endoscopy in adults [9] and is more frequently associated with HP than in children [10]. The frequency of GIM in children related to HP-positive gastritis versus HP-negative gastritis is variable. Shabib et al. [11] reported a frequency of 42% in children with HP-positive gastritis versus 6% in children with HP-negative gastritis. However, Kato et al. [12] documented no difference in the presence of intestinal metaplasia between the study groups of children with and without HP infection. However, no children in a Brazilian cohort of 96 children with HP gastritis were found to have GIM [13].

HP infection causes inflammatory cell infiltration in the gastric mucosa, resulting in atrophy of the foveolar epithelium and long-term mucosal changes such as intestinal metaplasia, which are precursors of gastric cancer [13–15]. HP organisms seem to be the most important member of the gastric microbiota with the highest relative abundance when present, but when it is absent, the stomach has a diverse microbiota [16]. Proteobacteria, Firmicutes, Actinobacteria, Bacteroidetes, and Fusobacteria are the most abundant phyla in both HP-positive and HP-negative patients [16].

Reactive gastropathy represents the second most common cause for the occurrence of age-dependent mucosal alterations [17]. Primary duodenogastric reflux (DGER) could cause gastric mucosal lesions manifested as intestinal metaplasia histologically in children. DGER is probably an independent etiological factor and might play a synergistic role in the pathogenesis of gastric mucosal lesions along with gastric acid and HP infection [18]. Other causes that are associated with GIM in adults include high gastric pH, increased bile acid exposure, smoking [19], and gastric denervation after surgery for benign disease [20].

The diagnosis of intestinal metaplasia can have adverse clinical implications and should be made with caution in a child [21]. The association of GIM with adenoma/dysplasia/carcinoma progression is commonly encountered in adults but is rarely seen in children. Only 10% of gastric cancer cases are found in patients younger than 40 years of age [22]. It is very likely that time plays an important factor the progression of GIM to adenoma. The progression from intestinal metaplasia to gastric adenocarcinoma takes an average of about 7 years in adult studies [23]. Thus, by the time GIM undergoes neoplastic transformation, the patient would become an adult and, therefore, managed in the adult service [5, 10]. The malignant potential of GIM has been shown to vary based on histologic subtype, location, and extent of mucosal involvement [24, 25]. Adults with incomplete GIM subtype versus complete GIM subtype, diffuse involvement of the antrum and gastric body versus antrum alone, and greater than 20% extension of mucosal involvement between endoscopic sampling had a greater risk of gastric cancer [25, 26]. Family history of gastric cancer on initial evaluation was associated with increased risk of subsequent gastric cancer in adult patients [9, 23, 24].

Treatment, long-term consequences, and surveillance protocols of GIM are not well established in the pediatric population. Contrary to our first case presented, a case report of a 15-year-old with GIM located inside the cryptic antral epithelium showed complete resolution following 3 months of PPI treatment [27]. In adults, as a pragmatic behavior, yearly endoscopic evaluation would appear justified in all GIM patients with at least one of these conditions: (1) IM extension > 20% mucosal involvement between endoscopies; (2) the presence of incomplete type IM; (3) first-degree relative of gastric cancer patients; and (4) smokers [24]. Controversy exists regarding whether routine surveillance should be performed in individuals with GIM in low HP prevalence regions such as the United States [28]. In patients with few risk factors, surveillance every 2-3 years could be proposed [24].

We believe it is important to report pediatric patients with GIM and, more importantly, ensure a long-term follow-up into adulthood in order to better understand the natural history of this disease and early detection of dysplasia, should it occur. We presented herein an overview of the current knowledge on the detection and surveillance of patients with GIM. However, the treatment, long-term consequences, and surveillance protocols are not well stablished in pediatric patients likely due to the limited literature available and the need for consensus on the follow-up of this histopathological finding. At present, GIM is frequently disregarded in clinical practice or results in widely varying follow-up frequency or treatment. These uncertainties require further research in the pediatric population.

## References

[1] de Vries A. C., Haringsma J., Kuipers E. J. (2007). The detection, surveillance and treatment of premalignant gastric lesions related to *Helicobacter pylori*infection. *Helicobacter*.

[2] Morson B. C. (1955). Intestinal metaplasia of the gastric mucosa. *British Journal of Cancer*.

[3] Capelle L. G., de Vries A. C., Haringsma J. (2010). The staging of gastritis with the OLGA system by using intestinal metaplasia as an accurate alternative for atrophic gastritis. *Gastrointestinal Endoscopy*.

[4] Dixon M. F., Genta R. M., Yardley J. H. (1996). Classification and grading of gastritis: the updated Sydney system. *The American Journal of Surgical Pathology*.

[5] Dimitrov G., Gottrand F. (2006). Does gastric atrophy exist in children?. *World Journal of Gastroenterology*.

[6] Dinis-Ribeiro M., Areia M., De Vries A. C. (2012). Management of precancerous conditions and lesions in the stomach (MAPS): guideline from the European Society of Gastrointestinal Endoscopy (ESGE), European Helicobacter Study Group (EHSG), European Society of Pathology (ESP), and the Sociedade Portuguesa de Endoscopia Digestiva (SPED). *Endoscopy*.

[7] Elitsur Y., Triest W. E. (1997). Is duodenal gastric metaplasia a consequence of Helicobacter pylori infection in children?. *American Journal of Gastroenterology*.

[8] Kanemitsu T., Yao K., Nagahama T. (2017). Extending magnifying NBI diagnosis of intestinal metaplasia in the stomach: the white opaque substance marker. *Endoscopy*.

[9] Reddy K. M., Chang J. I., Shi J. M., Wu B. U. (2016). Risk of gastric cancer among patients with intestinal metaplasia of the stomach in a us integrated health care system. *Clinical Gastroenterology and Hepatology*.

[10] Riddell R. H. (1999). Pathobiology of Helicobacter pylori infection in children. *Canadian Journal of Gastroenterology & Hepatology*.

[11] Shabib S. M., Cutz E., Drumm B., Sherman P. M. (1994). Association of gastric metaplasia and duodenitis with Helicobacter pylori infection in children. *American Journal of Clinical Pathology*.

[12] Kato S., Nakajima S., Nishino Y. (2006). Association between gastric atrophy and Helicobacter pylori infection in Japanese children: a retrospective multicenter study. *Digestive Diseases and Sciences*.

[13] Carvalho M. A., Machado N. C., Ortolan E. V. P., Rodrigues M. A. M. (2012). Upper gastrointestinal histopathological findings in children and adolescents with nonulcer dyspepsia with helicobacter pylori infection. *Journal of Pediatric Gastroenterology and Nutrition*.

[14] Uemura N., Okamoto S., Yamamoto S. (2001). Helicobacter pylori infection and the development of gastric cancer. *The New England Journal of Medicine*.

[15] Filipe M. I., Potet F., Bogomoletz W. V. (1985). Incomplete sulphomucin-secreting intestinal metaplasia for gastric cancer. Preliminary data from a prospective study from three centres. *Gut*.

[16] Alarcón T., Llorca L., Perez-Perez G. (2017). Impact of the microbiota and gastric disease development by Helicobacter pylori. *Current Topics in Microbiology and Immunology*.

[17] Sonnenberg A., Genta R. M. (2015). Changes in the gastric mucosa with aging. *Clinical Gastroenterology and Hepatology*.

[18] Ma M., Chen J., Zhang Y.-Y., Li Z.-Y., Jiang M.-Z., Yu J.-D. (2008). Pathogenic effects of primary duodenogastric reflux on gastric mucosa of children. *Chinese Journal of Pediatrics*.

[19] Nakamura M., Haruma K., Kamada T. (2001). Duodenogastric reflux is associated with antral metaplastic gastritis. *Gastrointestinal Endoscopy*.

[20] Ohira M., Toyokawa T., Sakurai K. (2016). Current status in remnant gastric cancer after distal gastrectomy. *World Journal of Gastroenterology*.

[21] Weinberg A. G. (2012). The significance of small intestinal epithelium in gastric antral biopsies in children. *Pediatric and Developmental Pathology*.

[22] Kokkola A., Sipponen P. (2001). Gastric carcinoma in young adults. *Hepato-Gastroenterology*.

[23] Olaofe O. O., Sabageh D., Komolafe A. O. (2016). A review of the clinicopathologic characteristics of intestinal metaplasia in gastric mucosal biopsies. *Pan African Medical Journal*.

[24] Zullo A., Hassan C., Romiti A. (2012). Follow-up of intestinal metaplasia in the stomach: when, how and why. *World Journal of Gastrointestinal Oncology*.

[25] Rugge M., Meggio A., Pennelli G. (2007). Gastritis staging in clinical practice: the OLGA staging system. *Gut*.

[26] Silva S., Filipe M. I. (1986). Intestinal metaplasia and its variants in the gastric mucosa of portuguese subjects: a comparative analysis of biopsy and gastrectomy material. *Human Pathology*.

[27] Kalach N., Papadopoulos S., Asmar E. (2009). In french children, primary gastritis is more frequent than helicobacter pylori gastritis. *Digestive Diseases and Sciences*.

[28] Correa P., Piazuelo M. B., Wilson K. T. (2010). Pathology of gastric intestinal metaplasia: clinical implications. *American Journal of Gastroenterology*.

